# Supplementary material for: Landscape of Genome-Wide DNA Methylation of Colorectal Cancer Metastasis
Source: Cancers (Basel). 2020 Sep 22;12(9):2710. doi: 10.3390/cancers12092710 (PMC7564781; doi:10.3390/cancers12092710)
Supplement: Supplementary file 1 [file cancers-12-02710-s001.pdf]

Supplementary Materials

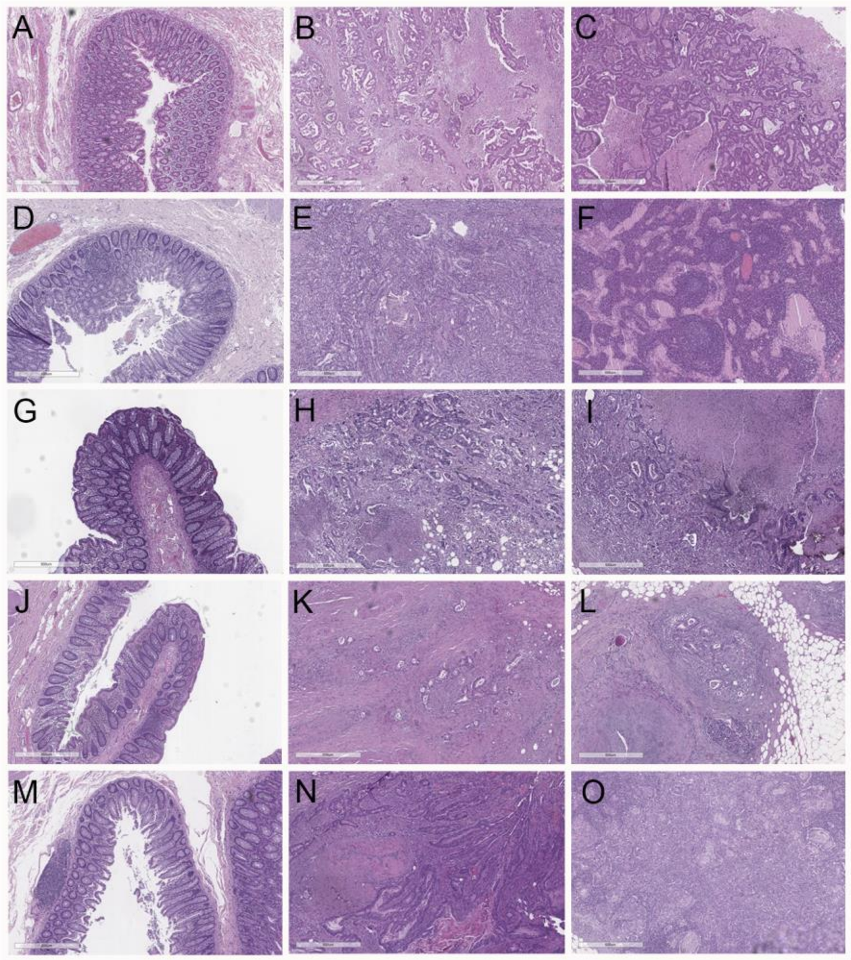

Figure S1. Representative histological sections stained with haematoxylin and eosin showing normal adjacent tissues (**A,D,G,J,M**), primary tumours (**B,E,H,K,N**) and lymph node metastasis (**C,F,I,L,O**) from Patient 1–5 respectively. Scale bar = 600 nm.

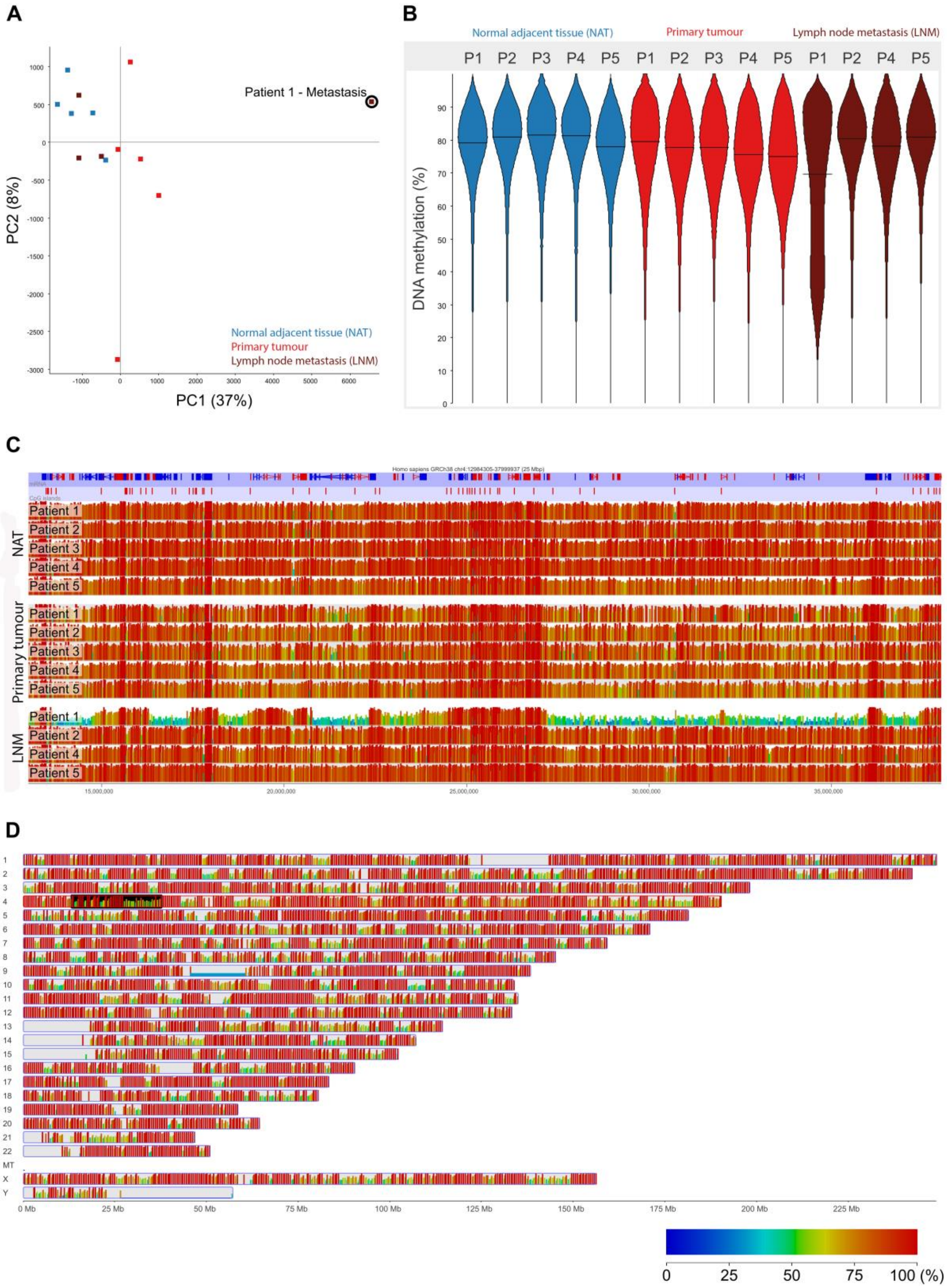

Figure S2. Quality control assessment identifying the metastasis sample of patient 1 (P1M) as outlier. (**A**) Principal component analysis shows that PC1, presenting 37% of total variation is driven by sample P1M. (**B**) Beanplots of methylation levels of 200 CpG windows for individual samples, show hypomethylation of sample P1M. The horizontal line indicates the median. Colour code: NAT = blue, primary tumour = red, LNM = burgundy. (**C**) Screenshot of a genome section identifies large hypomethylated regions in sample P1M. (**D**) Depiction of the whole genome shows that these hypomethylated blocks occur through the whole genome. B + C) Each bar represents a 200CpG window. Methylation levels are indicated by the height and colour of the bar. (NAT: normal adjacent tissue; LNM: lymph node metastasis).

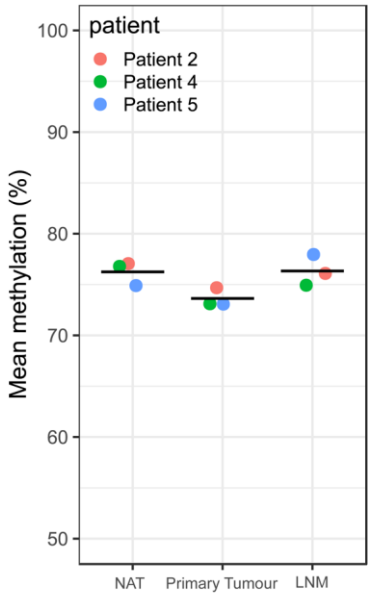

Figure S3. Global Methylation. Mean global methylation for each individual patient. The black line indicates the mean of the 3 patients.

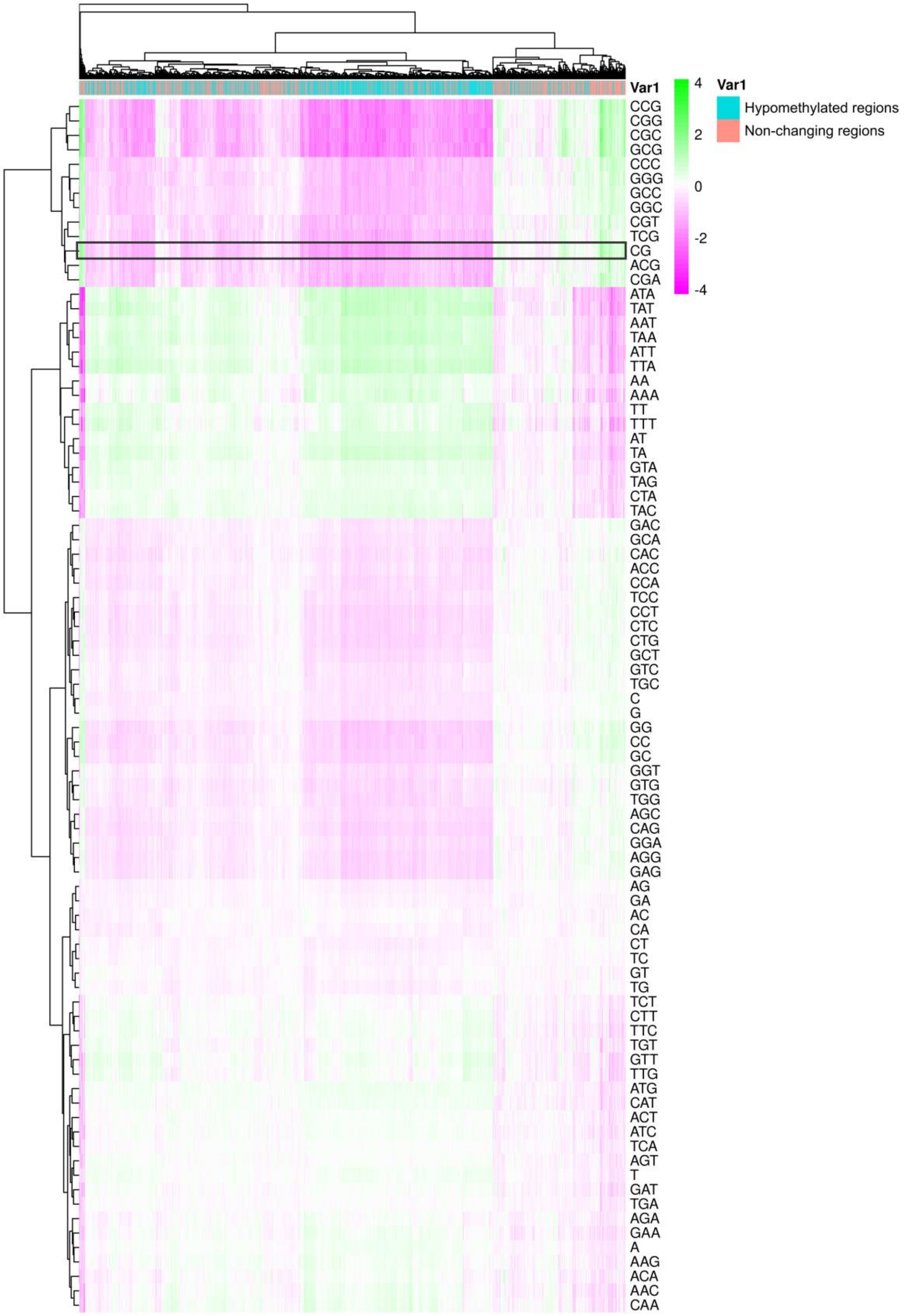

Figure S4. Sequence composition of primary tumour hypomethylated regions and non-changing regions. Clustered heatmap showing relative enrichment of k-mers for individual 200 CpG windows that are hypomethylated in primary tumour compared to NAT (blue) or that are not changing (orange), as indicated in the bar above. Each column represents a 200 CpG window and each row represents a different sequence. Level of enrichment is indicated by the colour. The row showing CG enrichment is highlighted by the black box.

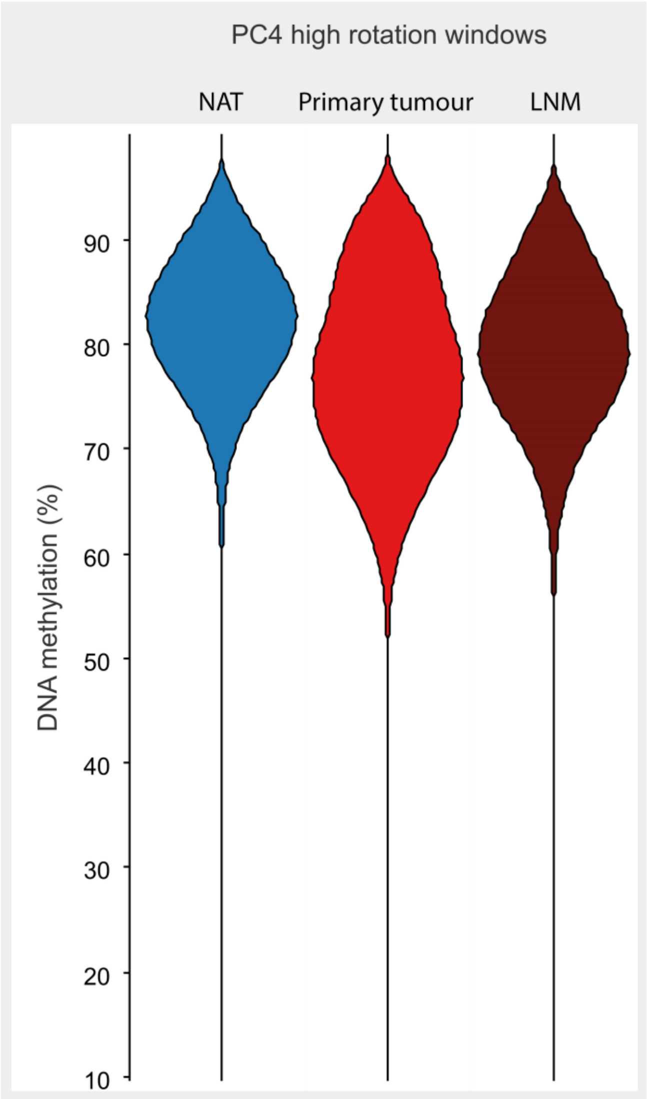

Figure S5. DNA methylation levels of probes underlying PC4 of principal component analysis from Figure 1.

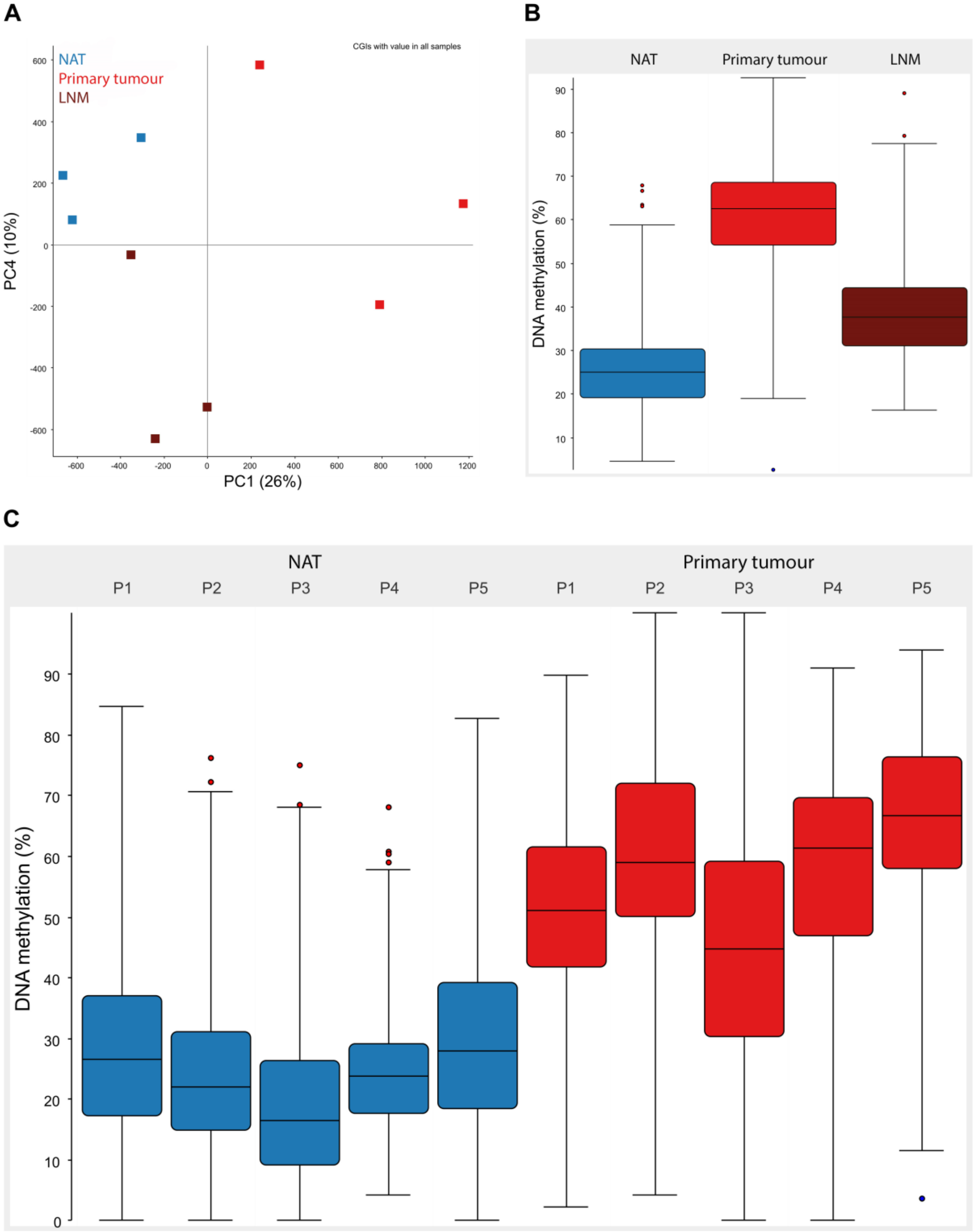

Figure S6. CGI methylation patterns and differentially methylated CGIs in normal colon epithelium, primary tumour and metastasis samples of CRC patients. (**A**) Principal component analysis of CGI methylation analysis split normal, primary tumour and metastasis samples into clusters on PC1 and PC4. B + C) Box-whisker plots showing DNA methylation levels of differentially methylated CGIs in normal, primary tumour and metastasis samples averaged from patients 2, 4 and 5 (**B**) and of normal and primary tumour samples of all individual patients (**C**). Differentially methylated CGIs were identified comparing normal and primary tumour samples of patients 1 to 5. Boxes represent the interquartile range; lines the median; dots beyond the whiskers, outliers.

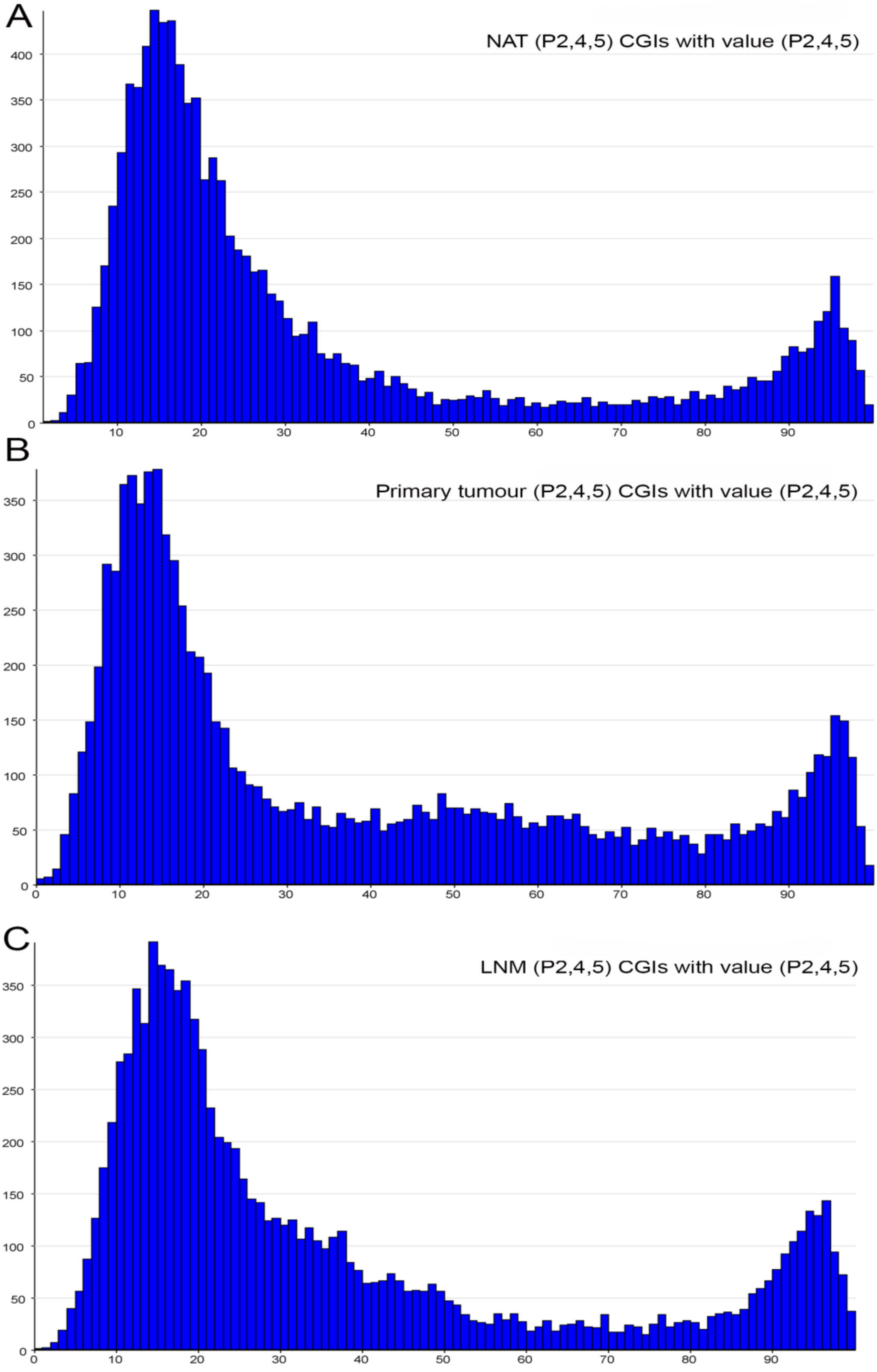

Figure S7. Probe value histograms of average of Patient 2, 4 and 5, showing the number of probes (= CGIs) on y-axis with a certain methylation levels, as indicated on x-axis. (**A**) NAT, (**B**) primary tumour and (**C**) LNM, showing the bimodal methylation pattern of CGIs. Whereas the majority of CGIs is lowly methylated some CGIs are very highly methylated.

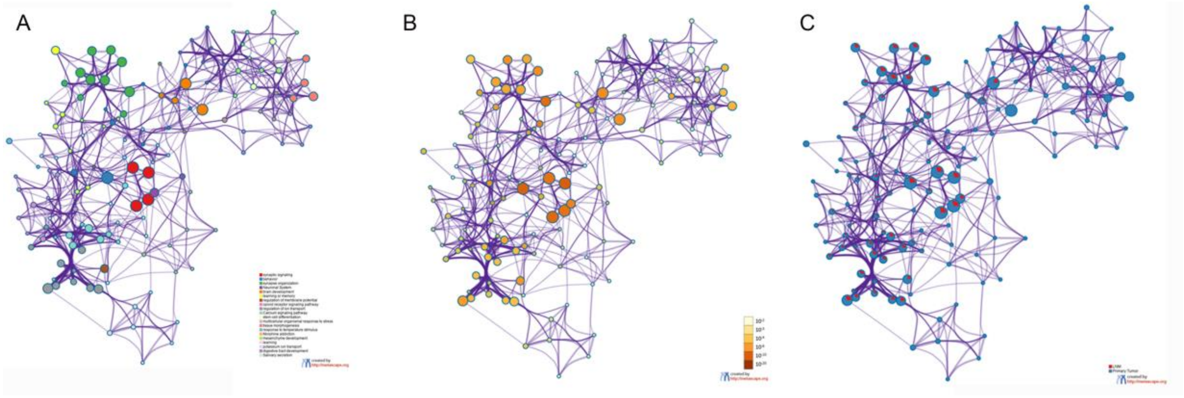

Figure S8. Enriched Ontology Clusters. (**A**) Network layout of enriched ontology coloured by cluster ID generated by Metascape. Each term is represented by a circle node, where its size is proportional to the number of input genes fall into that term, and its colour represent its cluster identity. Terms with a similarity score > 0.3 are linked by an edge (the thickness of the edge represents the similarity score). (**B**) The same enrichment network has its nodes coloured by *p*-value, as shown in the legend. (**C**) The same enrichment network has its nodes displayed as pies. Each pie sector is proportional to the number of hits originated from a gene list. Colour code for pie sector represents a gene list.

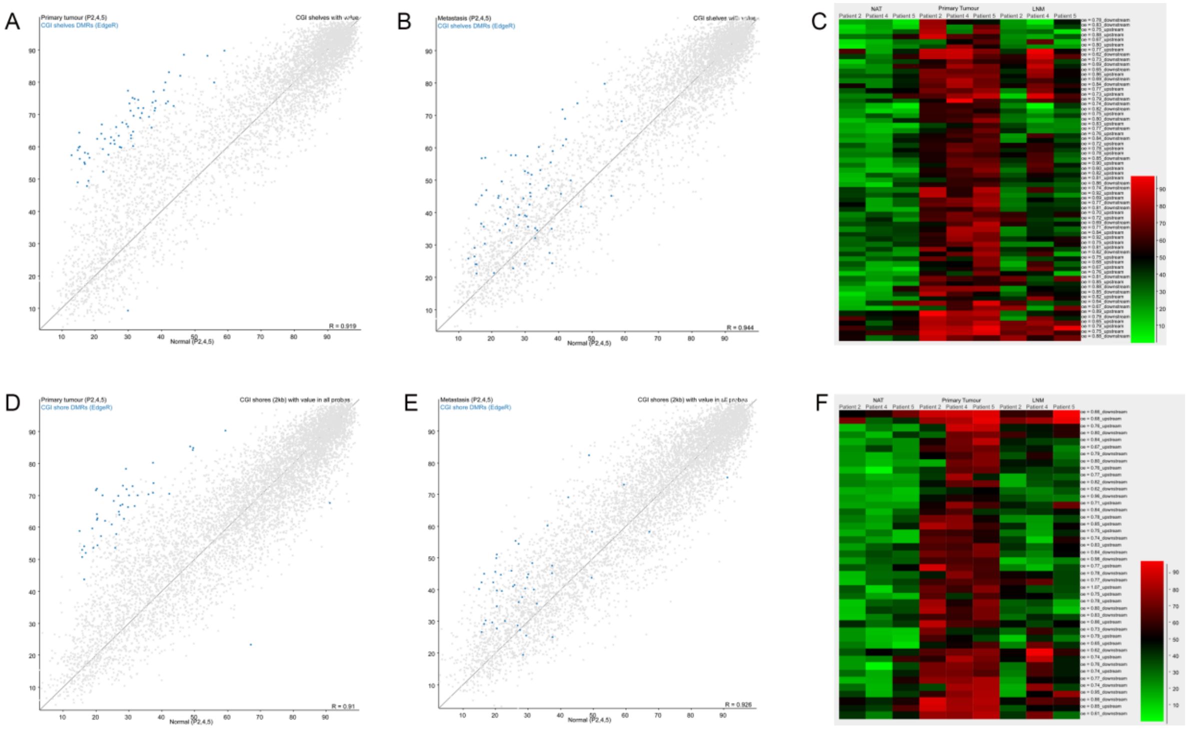

Figure S9. CGI shelves and shore analysis. (**A**) Scatterplot comparing DNA methylation levels of CGI shelves between NAT and PT samples. DMRs are highlighted in blue. (**B**) Scatterplot comparing DNA methylation levels of CGI shelves between NAT and LNM samples. DMRs (NAT vs PT) are highlighted in blue. (**C**) Heatmap showing methylation levels of individual samples (NAT, PT, LNM of P2, P4, P5) of 65 hypermethylated CGI shelves. (**D**) Scatterplot comparing DNA methylation levels of CGI shores between NAT and PT samples. DMRs are highlighted in blue. (**E**) Scatterplot comparing DNA methylation levels of CGI shores between NAT and LNM samples. DMRs (NAT vs PT) are highlighted in blue. (**F**) Heatmap showing methylation levels of individual samples (NAT, PT, LNM of P2, P4, P5) of 44 hypermethylated CGI shores. DMR: Differentially methylated regions; NAT: Normal adjacent tissue; PT: Primary tumour; LNM: Lymph node metastasis).
